# Augmented Reality-Assisted Microsurgery for Severe Thoracic Ossification of the Ligamentum Flavum: A Case Report

**DOI:** 10.7759/cureus.76063

**Published:** 2024-12-20

**Authors:** Shinji Saito, Shunsuke Katsumi, Akira Shinohara, Daigo Arimura, Mitsuru Saito

**Affiliations:** 1 Department of Orthopaedic Surgery, The Jikei University School of Medicine, Tokyo, JPN

**Keywords:** augmented reality, elastic image fusion, microscopy, navigation, ossification of the ligamentum flavum

## Abstract

Ossification of the ligamentum flavum (OLF) can lead to dural ossification, significantly increasing the risk of complications, including intraoperative nerve injury. The application of augmented reality (AR) and advanced digital technologies in spine surgery has the potential to reduce these risks. This case report highlights a perioperative nerve injury-free microsurgery using elastic image fusion technology, which integrates preoperative imaging with intraoperative computed tomography for a patient with severe stenotic OLF and dural ossification.

A 68-year-old Japanese man presented with persistent right-sided back pain. Additionally, the patient reported mild gait instability and difficulty maintaining balance on uneven surfaces, which had progressively worsened over the past six months. Magnetic resonance imaging revealed severe OLF with 81% spinal canal stenosis. Given the risks of dural injury and cerebrospinal fluid leakage, a microsurgical procedure using AR was planned to ensure nerve protection. The surgery employed an image-guided navigation system with elastic image fusion to accurately align intraoperative and preoperative images. Additionally, microscopy enabled the real-time projection of preoperative images and navigation screens onto the surgical field. The procedure was successful, and the patient experienced no postoperative nerve damage. He regained walking stability and was discharged on the 28th postoperative day. At the two-year follow-up, he remained free of recurrences and neurological deficits.

OLF with dural involvement poses a high risk of complications. In such complex cases, AR technology provides valuable intraoperative reference information, enhancing the safety and precision of spinal surgery.

## Introduction

With the widespread use of computed tomography (CT) and magnetic resonance imaging (MRI), the diagnosis of ossification of the ligamentum flavum (OLF) has become relatively easy, and its prevalence is consequently increasing [1]. Advanced OLF can cause dural ossification and carries a high risk of complications, such as intraoperative nerve damage [2]. Augmented reality (AR) and other surgical digital technologies are advancing in the field of spine surgery and are helping to reduce the risk of complications [3,4]. Here, we report a case of perioperative nerve injury-free microsurgery using elastic image fusion, which combines preoperative images with intraoperative CT images in spinal surgery for severe stenotic OLF with dural ossification.

## Case presentation

**Figure 1:**
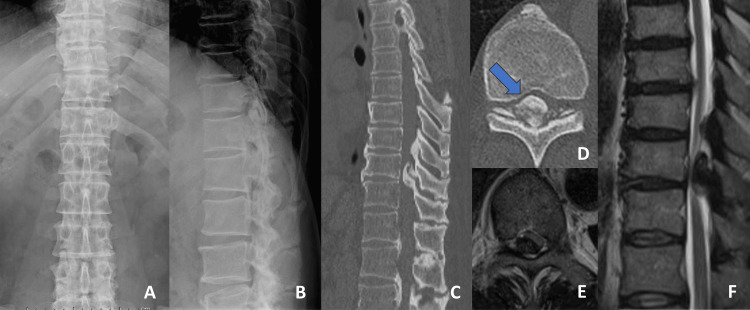
Preoperative X-ray, computed tomography (CT), and magnetic resonance imaging (MRI). (A) X-ray: anteroposterior view. (B) X-ray: lateral view. (C) Sagittal CT. (D) Axial CT: severe OLF with an occupation rate of 81%, classified as the tuberous type (arrow). (E) T2-weighted axial MRI. (F) T2-weighted sagittal MRI.

A 68-year-old Japanese man presented with a complaint of right-sided back pain. He had first noticed the pain six months prior and had sought medical attention locally. Apart from the right-sided back pain, he had no other symptoms and no objective findings, including neurological deficits. However, due to the lack of improvement in his pain, an MRI was performed, which revealed severe OLF with a spinal canal occupancy rate of 81%. The patient was subsequently referred to our institution for further management. Laboratory tests, including blood biochemistry, showed no significant abnormalities on admission. Physical examination at the initial visit revealed spontaneous pain in the right back, but no tenderness to percussion. The patient did not experience intermittent claudication, and both the straight leg raise test and the femoral nerve stretch test were negative. Additionally, the Kemp sign was not observed. Neurological examination revealed hyperreflexia in both the patellar tendon reflex and the Achilles tendon reflex. Additionally, the patient reported gait instability and noted an increased tendency to fall. Muscle strength testing was performed, with the results indicating no reduction in muscle strength in the iliopsoas, quadriceps, tibialis anterior, extensor hallucis longus, flexor hallucis longus, gastrocnemius, or hamstring. No sensory impairments were observed, and the patient did not report any bladder or rectal dysfunction. Imaging studies revealed a bony OLF, protruding as a mass on the posterior edge of the intervertebral foramen, as seen on the lateral view of the radiographs. CT scan revealed severe OLF with an occupation rate of 81%, classified as a tuberous type according to Sato et al. [5], suggesting a poor prognosis [6]. The OLF extended from T9/10 to T10/11, with the most severe stenosis observed at T10/11. Although the MRI showed significant spinal cord compression due to narrowing, no clear intramedullary signal intensity changes were observed (Figure 1).

**Figure 2:**
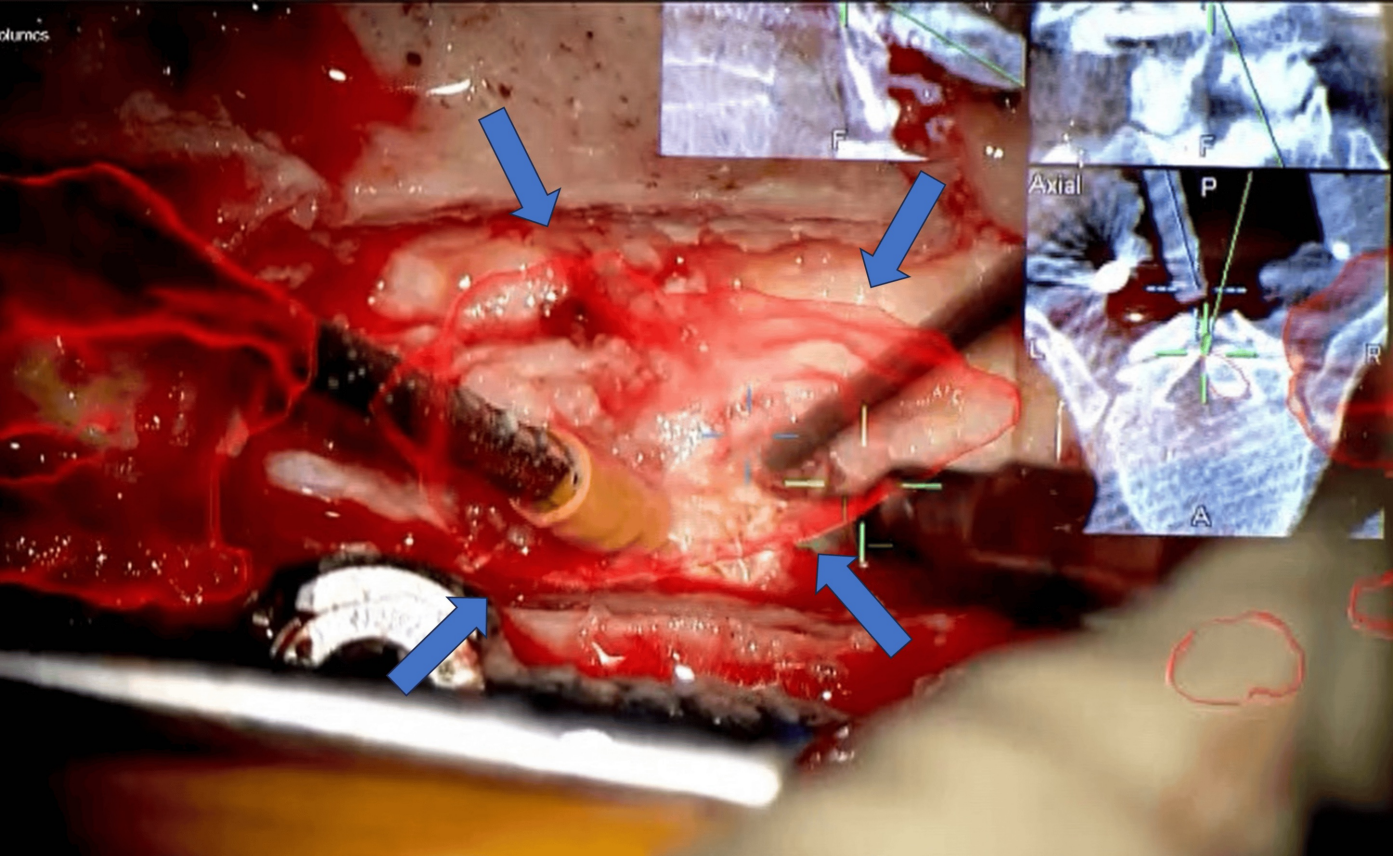
Intraoperative microscope screen. The preoperative image of the ossified lesion and the navigation screen can be projected onto the same display as the surgical field. The red box indicated by the arrow highlights the ossified lesion. The upper-right corner shows the navigation screen, allowing for the simultaneous confirmation of the depth of the ossification.

We planned a posterior spinal decompression fixation of T9-12. By utilizing AR technology to visualize the extent and depth of ossification, we decided to proceed with total resection of the OLF. After intraoperative CT using Artis Pheno (Siemens Healthineers AG, Forchheim, Germany), pedicle screws were inserted into T9-12 using the Curve automatic registration navigation system (Brainlab headquarters, München, Germany). A microscope PENTERO (Carl Zeiss Meditec AG, Oberkochen, Germany) was linked to the navigation system to confirm the position of the screw (Figure 2).

**Figure 3:**
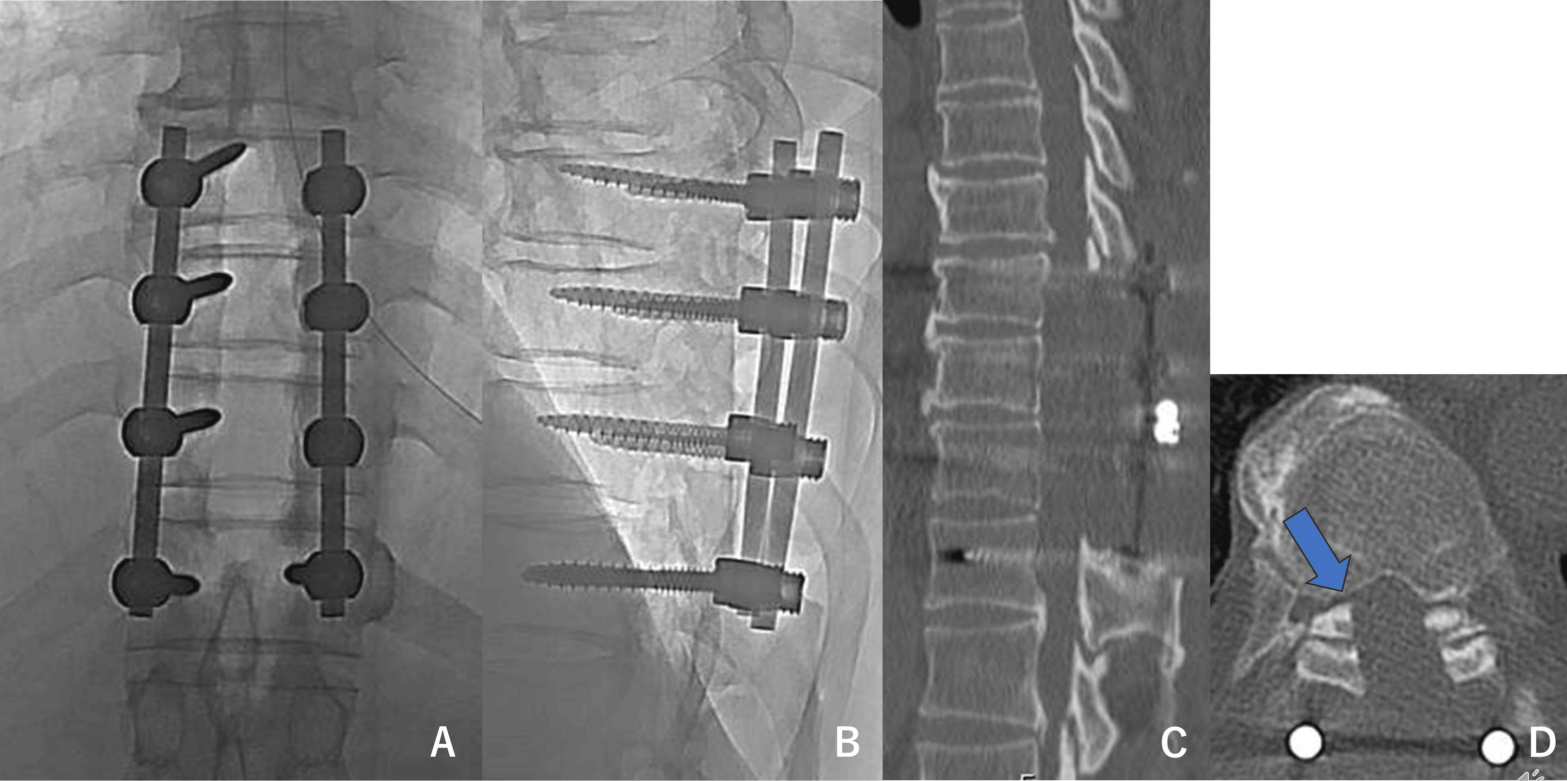
Postoperative X-ray and computed tomography (CT). (A) X-ray: anteroposterior view. (B) X-ray: lateral view. (C) Sagittal CT. (D) Axial CT: all ossifications of the ligamentum flavum were resected (arrow).

The most severe stenosis, located at the T10/11 level, was excavated using an ultrasonic aspirator. Using the navigation system, the ossified lesion was identified and carefully thinned until it resembled an eggshell. The ossified dura mater was then floated and resected in a single piece while detaching the adhesions. After resection of the ossified dura mater, dural suture and repair were performed for two dural injuries that were identified. Complete excision of the ossified lesion was confirmed on postoperative radiographs (Figure 3).

To prevent spinal fluid leakage, the patient started rehabilitation by transferring to a wheelchair before beginning to walk. The patient’s back pain was relieved before the surgery, and he has been doing well without any recurrence of ossification or neurological symptoms in the two years since the surgery.

## Discussion

According to Sato et al. [5], the ossification pattern in this case was the tuberous type. Sun et al. [6] reported that 86% of patients with the tuberous type have dural ossification. In addition, preoperative CT showed the tram track and comma signs, which are characteristic of dural ossification, with a sensitivity of 94.21% [7]. It has also been reported that a spinal canal occupancy rate of more than 55% suggests dural ossification [8]. In OLF surgery, dural injury and cerebrospinal fluid leakage occur as frequently as 32% [2] and are expected to occur even more frequently in patients with dural ossification. Therefore, we elected to perform the surgery using intraoperative CT with the Curve navigation system.

AR is commonly classified under the umbrella term “extended reality,” alongside virtual reality (VR) and mixed reality (MR). However, AR and VR are often conflated. VR technology allows users to become immersed in a computer-generated virtual space using a headset or goggles. In contrast, AR technology superimposes virtual information onto the real world, typically displayed on a screen through devices such as a smartphone or goggles. While VR creates a virtual environment, AR enhances the real world as viewed through a screen. Recently, the potential application of AR in health care has been attracting significant attention, with AR being adopted in educational settings and as an aid during surgical procedures in the clinical environment. AR has begun to be introduced in spinal surgery to enhance the understanding of complex anatomy and is also suggested to be beneficial in resident education [9].

In neurosurgical craniotomy, the position of the patient before and during surgery does not change significantly, but in the field of spine surgery, the alignment of the vertebrae changes, making the clinical application of AR technology difficult. In this study, we used the Curve automatic registration navigation system, which has an elastic image fusion function to eliminate errors between the intraoperative and preoperative positions. Conventional AR systems fuse images for each vertebral body, but elastic image fusion corrects vertebral body alignment by allowing separate images to be fused, even for lesions that span other vertebral bodies. By extracting and marking the ossified lesion from preoperative CT images, this function allows for the synthesis of intraoperative CT images. The microscope system used in this case can project the preoperative image of the ossification lesion and the navigation screen onto the same screen as the operative field. While confirming the extent of the ossification, the depth of the ossification can be simultaneously confirmed on the navigation screen, allowing the surgeon to reach the ossification by the shortest route and successfully remove it. The spinal canal occupancy in the present case was 81%, and extensive ossification of the dura mater was observed. Adhesion between the ossification and the dura mater was also observed, and two dural injuries were encountered during the dissection of the ossification. The accuracy of the preoperative and intraoperative CT image data was reported by Brainlab to have a median error of 1.34 mm [10]. Although the accuracy is still inadequate for spine surgery, where accuracy to the nearest 1 mm is essential, we believe that having a large volume of intraoperative reference information is useful for ensuring the smooth and safe performance of a surgical procedure.

## Conclusions

The surgical management of severe stenosis OLF with dural ossification is a challenge due to the high risk of complications. AR technology enables real-time localization and depth perception of the ossification without disrupting the surgical field of view. Although it remains necessary to improve the accuracy of image data, AR technology can provide a wealth of intraoperative reference information, making it a valuable tool for enhancing the safety of spinal surgery.
